# Combined Association of Vitamin D and Sex Hormone Binding Globulin With Nonalcoholic Fatty Liver Disease in Men and Postmenopausal Women: A Cross-Sectional Study

**DOI:** 10.1097/MD.0000000000002621

**Published:** 2016-01-29

**Authors:** Ningjian Wang, Hualing Zhai, Chaoxia Zhu, Qin Li, Bing Han, Yi Chen, Chunfang Zhu, Yingchao Chen, Fangzhen Xia, Dongping Lin, Yingli Lu

**Affiliations:** From the Institute and Department of Endocrinology and Metabolism, Shanghai Ninth People's Hospital, Shanghai JiaoTong University School of Medicine, Shanghai, China (NW, HZ, QL, BH, YC, CZ, YC, FX, DL, YL); Department of Endocrinology and Metabolism, The First Affiliated Hospital of Henan University of Science and Technology, Henan, China (CZ).

## Abstract

This study aimed to explore the combined associations of 25(OH)-vitamin D and sex hormone binding globulin (SHBG) with nonalcoholic fatty liver disease (NAFLD) in men and postmenopausal women.

Our data, which were based on the population, were collected from 16 sites in East China in 2014. There were 2700 men with a mean age of 53 years and 1461 women over 55 who were considered postmenopausal enrolled in the study. Levels of 25(OH)D and SHBG were measured using chemiluminescence assay. NAFLD was measured using liver ultrasound. Multivariable-adjusted logistic regression models examined associations of 25(OH)D and SHBG tertiles with odds of mild and moderate–severe NAFLD.

Both the low 25(OH)D and low SHBG groups were significantly associated with higher odds of mild NAFLD (men: OR 1.37, 95% CI 1.05, 1.78 in low 25(OH)D group; OR 1.73, 95% CI 1.23, 2.45 in low SHBG group; women: OR 1.51, 95% CI 1.08, 2.12 in low 25(OH)D group; OR 2.16, 95% CI 1.48, 3.14 in low SHBG group) and moderate–severe NAFLD (men: OR 1.61, 95% CI 1.24, 2.10 in low 25(OH)D group; OR 3.42, 95% CI 2.41, 4.87 in low SHBG group; women: OR 1.66, 95% CI 1.14, 2.42 in low 25(OH)D group; OR 6.84, 95% CI 4.31, 10.84 in low SHBG group). However, the combined association of low 25(OH)D and low SHBG was much larger, especially in moderate–severe NAFLD (men: OR 6.57, 95% CI 3.87, 11.18; women: OR 8.16, 95% CI 3.98, 16.73). The associations were independent of age, total testosterone, abdominal obesity, diabetes, and lipid profile.

The negative associations of 25(OH)D and SHBG levels with NAFLD are strongest when viewed in combination in men and postmenopausal women. Further studies should determine the cause–effect relationship and investigate the underlying mechanisms of this finding.

## Abstract 2

Supplemental Digital Content is available in the text

## INTRODUCTION

Nonalcoholic fatty liver disease (NAFLD) is a state of fat accumulation in the liver that is not induced by alcohol abuse.^1^ Because of the high prevalence and risk of nonalcoholic steatohepatitis, and even cirrhosis seen in recent decades, NAFLD has become a public health problem of great importance.^2^ NAFLD increases the risk for obesity, diabetes mellitus, metabolic syndrome, and mortality related to cardiovascular disease.^3,4^ This suggests that NAFLD is a distinct clinical component of overall metabolic health.

Recently, it has been reported that sex hormone binding globulin (SHBG) and vitamin D are 2 novel factors associated with NAFLD, diabetes, and cardiovascular disease.^5–8^ Vitamin D exerts a classical function on calcium/phosphorus homeostasis, but it has also been reported to affect the function of the immune system, cell differentiation and proliferation, etc.^6^ Vitamin D deficiency is often found together with NAFLD, and this finding is not unexpected because it may influence NAFLD through mechanisms such as hepatic endotoxin exposure and profibrotic effects.^9^

SHBG, a serum-steroid transporting protein, is mainly synthesized in the liver. After being secreted into the blood, it binds sex hormones, transports them to target tissues and regulates their biological activities.^7^ Recent novel insights indicate that a reduction in SHBG level seems to be the convergence of crosstalk among inflammation, diabetes, obesity, and the risk for cardiovascular diseases.^7^ Furthermore, in 1 national study, men in the high tertile of SHBG were 54% less likely to have NAFLD than in the lowest tertile.^5^ It also has been noted that liver fat but not total body or visceral fat is significantly associated with SHBG levels.^10^

Why do the Chinese have such a high prevalence of NAFLD, which could be up to 40% in the general population?^11^ Studies often consider vitamin D and SHBG individually; however, these factors are closely linked. Two recent studies have found that vitamin D was positively related with SHBG levels.^12,13^ Is NAFLD associated with the high prevalence of vitamin D deficiency in China^13^ or the possible important role of SHBG in metabolic diseases^7^ or their combined effect? These questions are why we started to look into the association among those three factors. However, there is no evidence of their combined association with NAFLD. Furthermore, the relative importance of SHBG and vitamin D combinations in relation to the risk of NAFLD has not been investigated within a single analytical framework.

A large investigation, the Survey on Prevalence in East China for Metabolic Diseases and Risk Factors (SPECT-China), was performed in 2014. Based on the resulting data, the present study aimed to clarify the associations by investigating the NAFLD risk among men and postmenopausal women with different levels and combinations of SHBG and vitamin D.

## METHODS

### Participants

SPECT-China is a cross-sectional survey in East China (ChiCTR-ECS-14005052, www.chictr.org.cn).^14,15^ Chinese citizens ≥18 years old who had lived in their current area for ≥6 months were selected. We also excluded subjected with severe communication problems, acute illness or who were unwilling to participate. Between February 2014 and June 2014, 6899 subjects who were 18 to 93 years old were recruited in the SPECT-China study from 16 sites in Shanghai, Zhejiang, and Jiangxi Province.^14,15^ Detailed sampling information was described in a previous study.^14^ The study was approved by the Ethics Committee of Shanghai Ninth People's Hospital, Shanghai JiaoTong University School of Medicine. All patients to be included signed the informed consent.

For women in this study, we selected women over 55 who were considered postmenopausal, in accordance with previous studies.^15–17^ In China, at 55 years old, 97% of women are postmenopausal.^15^ Because the SHBG may be fluctuating with estradiol in the premenopausal women,^18^ it may not be appropriate to combine all premenopausal women into a single group for analysis.

**FIGURE 1:**
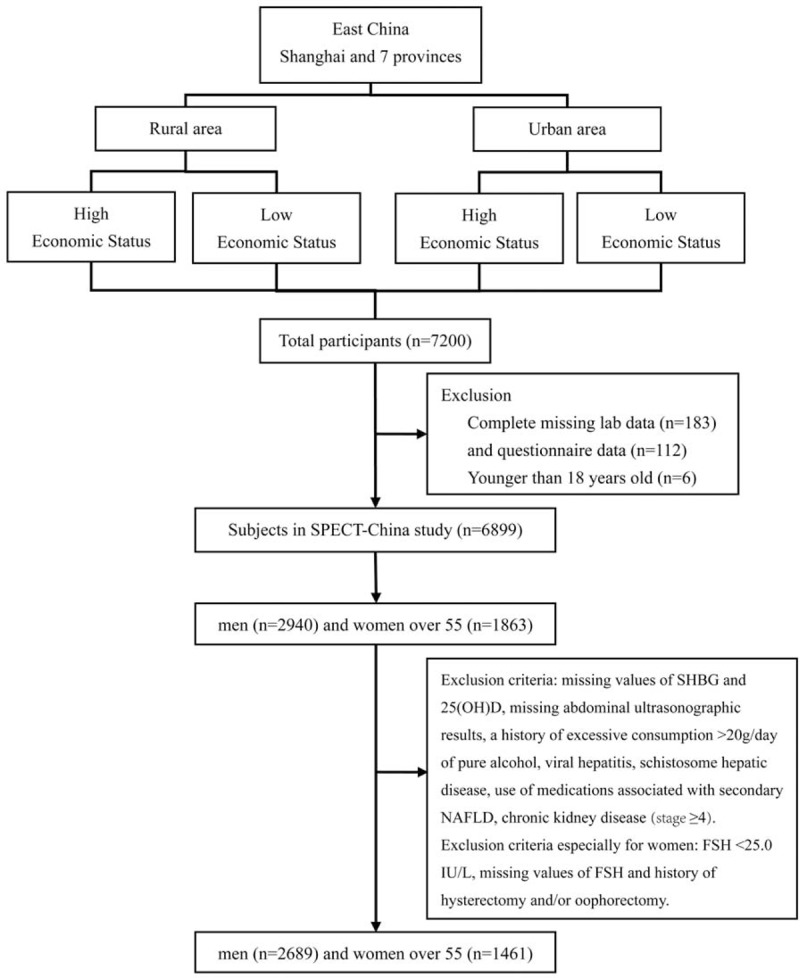
Flowchart of the sampling design and participants selected from SPECT-China.

There were 2940 men included. Men were excluded who had missing values of SHBG (n = 79) and 25-hydroxy-vitamin D (25(OH)D) (n = 1), were without abdominal ultrasonographic results (n = 114), or had a history of excessive consumption >20 g/day of pure alcohol (n = 17), viral hepatitis (n = 29), schistosome hepatic disease (n = 1), medications related to NAFLD (corticosteroids, amiodarone, methotrexate) (n = 6), or chronic kidney disease (stage ≥4) (n = 4). There were 1863 women who were older than 55, not using hormone replacement therapy and had no history of excessive consumption (>20 g/day) of pure alcohol. Exclusion criteria included missing values of SHBG (n = 174) or 25(OH)D (n = 1), follicle-stimulating hormone (FSH) <25.0 IU/L (according to the 2011 Stages of Reproductive Aging Workshop +10 recommendation,^19^ late perimenopausal state is characterized as FSH level ≥25 IU/L) (n = 42), missing values of FSH (n = 6), missing abdominal ultrasonographic results (n = 120), history of hysterectomy and/or oophorectomy (n = 24), self-reported viral hepatitis (n = 19), schistosome hepatic disease (n = 1), treatment with medications related to NAFLD (corticosteroids, tamoxifen, amiodarone, methotrexate) (n = 12) and chronic kidney disease (stage ≥4) (n = 4). Finally, the present study included 2689 men and 1461 women (Figure 1).

### Measurements

Participants fasted for 8 hours before the investigation. Blood samples were obtained between 7:00 am and 10:00 am. The blood samples for the fasting plasma glucose (FPG) were centrifuged within 1 hour after collection. Other blood samples were shipped in dry ice within 2 to 4 hours of collection to a laboratory that is certified by the College of American Pathologists. The 25(OH)D (Siemens ADVIA Centaur XP, Germany), total testosterone, FSH (Siemens, IMMULITE 2000, Erlangen, Germany) and SHBG levels (Roche Cobas E601, Basel, Switzerland) were detected using a chemiluminescence assay. Glycated hemoglobin (HbA1c) was measured by high-performance liquid chromatography (MQ-2000PT, Medconn, Shanghai, China). Plasma glucose, alanine aminotransferase (ALT), triglycerides, high-density lipoprotein (HDL) and low-density lipoprotein (LDL) were measured by a Beckman Coulter AU 680 (Brea, USA). The interassay and intraassay coefficients of variation were 6.6% and 5.7% for total testosterone, 4.5% and 3.8% for FSH, and 7.0% for SHBG.

### Clinical and Anthropometric Measurements

Same trained staff used a questionnaire to collect data about demography, medical history, and lifestyle risk factors at each site. Weight and height were measured using a weight balance and a vertical ruler when subjects wore light clothing without shoes. Body mass index (BMI) was calculated as weight in kilograms divided by height in meters squared. Waist circumference was measured at a level midway between the lowest rib and the iliac crest. Blood pressure was measured using standard methods as described previously.^20^

### Definition of Variables

Two experienced ultrasonographers from the same clinic setting used an ultrasound device (MINDRAY M7, Shenzhen, China) to perform an abdominal ultrasonographic examination. They did not know the study objective and were blinded to laboratory values. The diagnostic criteria for fatty liver by ultrasonography included “increased liver echogenicity, stronger echoes in the hepatic parenchyma than in the renal parenchyma, vessel blurring and narrowing of the lumen of the hepatic veins.”^21–23^ Based on the criteria by Saadeh et al,^21,22^ the degree of fatty liver on ultrasonography was categorized into normal, mild, and moderate–severe groups. According to the American Diabetes Association in 2014, the presence of diabetes was determined when a previous diagnosis had been made by a healthcare professional, FPG ≥7.0 mmol/L or HbA1c ≥6.5%. Abdominal obesity was defined as a waist circumference ≥90 cm in males and ≥80 cm in females.^20^ The diagnosis of metabolic syndrome was considered based on the International Diabetes criteria.^24^

### Statistical Analysis

Statistical analyses were performed using IBM SPSS Statistics, Version 22 (IBM Corporation, Armonk, NY). All analyses were 2-sided. A *P*-value <0.05 indicated significance. Continuous and categorical variables were expressed as the mean ± standard deviation (SD) and a percentage (%), respectively. To test for differences of characteristics among different levels of NAFLD, SHBG, and 25(OH)D, the Kruskal–Wallis test and 1-way ANOVA were used for continuous data with skewed and normal distributions, and the Pearson χ^2^ test was used for categorical variables.

SHBG and 25(OH)D were divided into tertiles, with the first tertile representing the lowest one and the third tertile the highest, using the third tertile as the reference. Logistic regression models were used to obtain the odds ratios (ORs) with 95% confidence intervals (CIs) as estimates of the associations between SHBG and 25(OH)D, separately and in combination, with NAFLD. Estimates were first adjusted for age and testosterone levels (model 1), and then they were further adjusted for abdominal obesity, diabetes, HDL, LDL, and triglycerides (model 2). A statistical interaction between SHBG and 25(OH)D was tested by adding a multiplicative factor in the logistic regression models.

Sensitivity analyses were performed. We showed the Spearman correlation between metabolic factors and the 25(OH)D and SHBG levels in continuous variables. Additionally, the logistic analyses are shown for NAFLD ORs when 25(OH)D and SHBG levels were included as continuous variables. The average age of the men examined was approximately 50 to 60 years, which may be representative of later adulthood, so we also analyzed the association in men younger than 50. We further explored the association of SHBG and 25(OH)D in combination with metabolic syndrome.

## RESULTS

### Sample Characteristics

The study sample included 2689 men with a mean age of 53(SD 13) years and 1461 postmenopausal women. As shown in Table 1, the prevalence of mild and moderate–severe NAFLD was 22.0% and 32.1%, respectively, in men and 22.6% and 25.9%, respectively, in women. Men and women with moderate–severe NAFLD were relatively younger but had significantly greater SHBG, 25(OH)D, ALT, blood pressure, LDL, and triglyceride levels (all *P* < 0.05). They also had a significantly higher prevalence of diabetes, abdominal obesity, and metabolic syndrome (all *P* < 0.05). In Supplemental Table S1, we also found that compared with men, postmenopausal women had significantly higher SHBG and lower 25(OH)D, ALT, and waist circumference (age-adjusted *P* < 0.05).

**TABLE 1:**
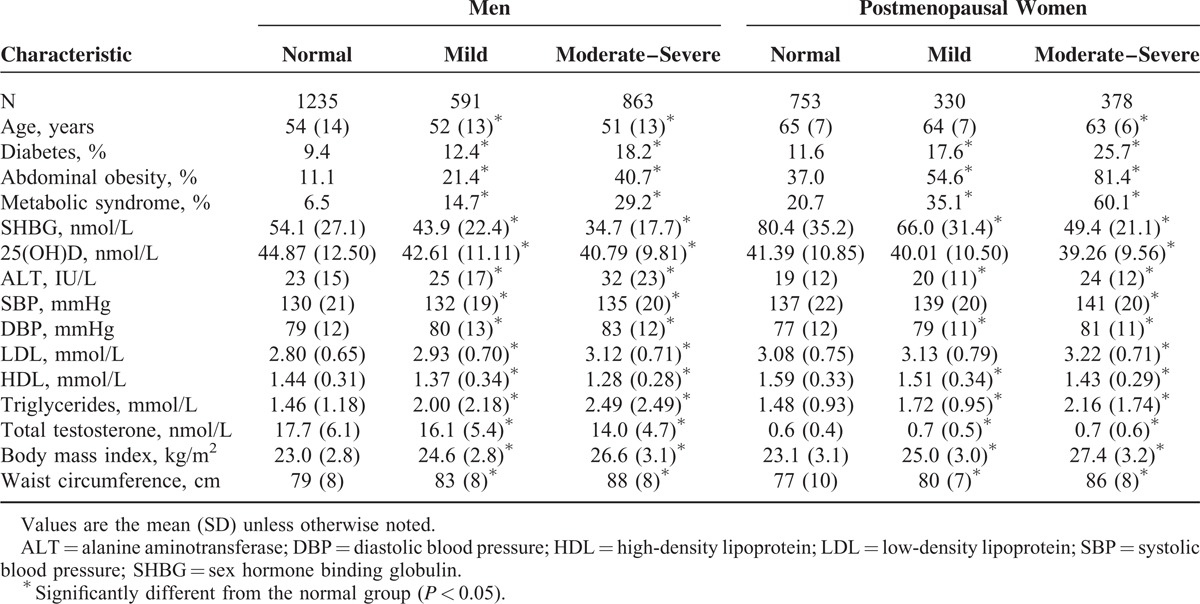
Characteristics of Participants by Degrees of Hepatic Steatosis (N = 4150)

In Table 2, we observed that the prevalence of mild and moderate–severe NAFLD in the low 25(OH)D and low SHBG group was significantly greater than that in the high 25(OH)D and high SHBG group in men and women (*P* < 0.05).

**TABLE 2:**
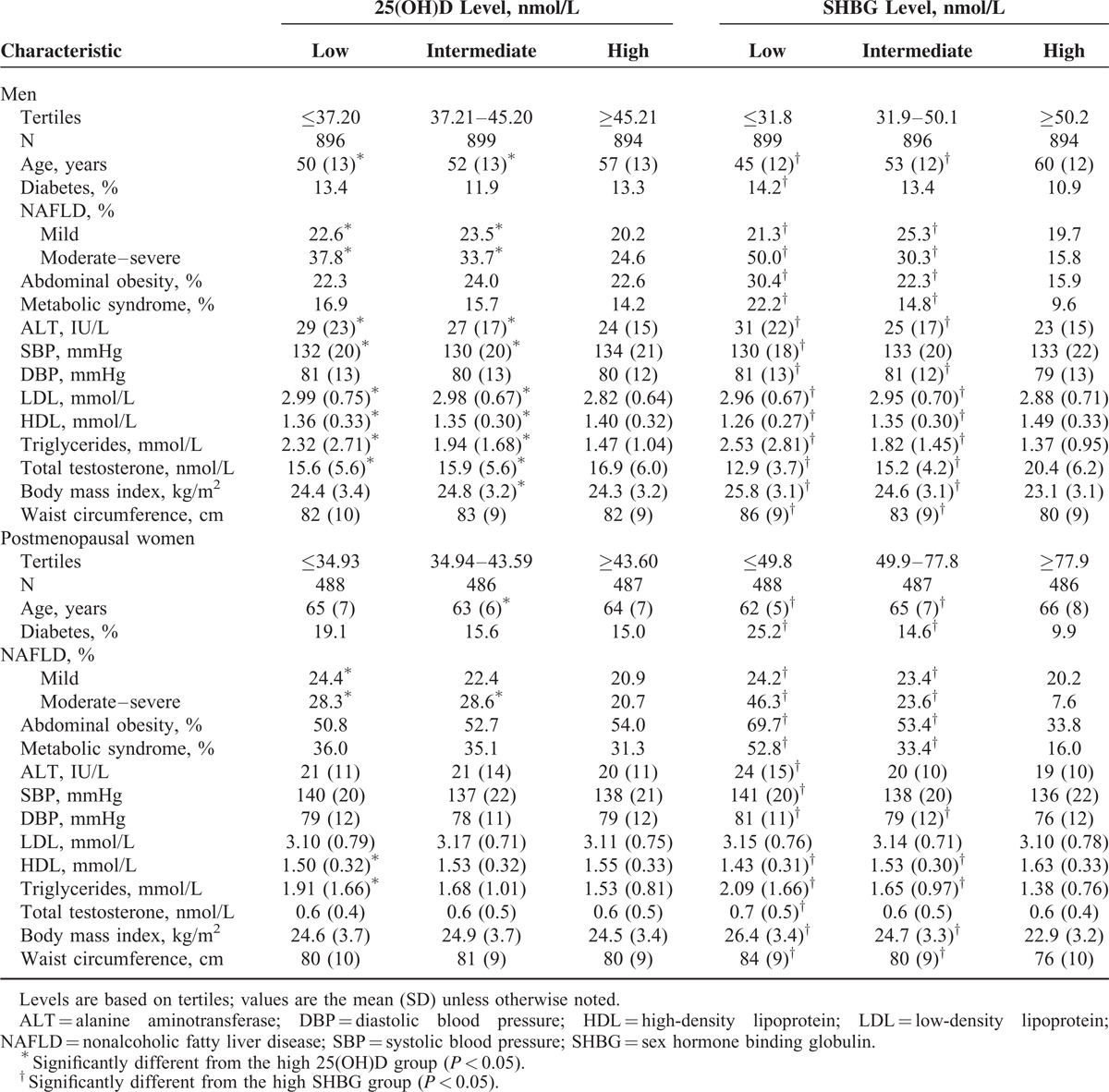
Characteristics of Participants by Tertiles of 25(OH)D and SHBG (N = 4150)

### The Separate Association of 25(OH)D and SHBG With NAFLD

As shown in Table 3, compared with having a high level of 25(OH)D, having a low level was associated with 1.49 (95% CI 1.16, 1.91, men) and 1.45 (95% CI 1.05, 2.01, women) times higher odds of mild NAFLD and with 1.86 (95% CI 1.47, 2.36, men) and 1.70 (95% CI 1.21, 2.39, women) times higher odds of moderate–severe NAFLD in models adjusted for age and testosterone (model 1). Similarly, compared with having a high level of SHBG, having a low level was associated with 2.20 (95% CI 1.58, 3.07, men) and 2.91 (95% CI 2.07, 4.10, women) times higher odds of mild NAFLD, and with 5.02 (95% CI 3.66, 6.88, men) and 13.78 (95% CI 9.15, 20.73, women) times higher odds of moderate–severe NAFLD in model 1. In multivariable-adjusted models (model 2) that were further adjusted for abdominal obesity, diabetes, lipid profile, and systolic blood pressure, most of these associations were attenuated but still significant.

**TABLE 3:**
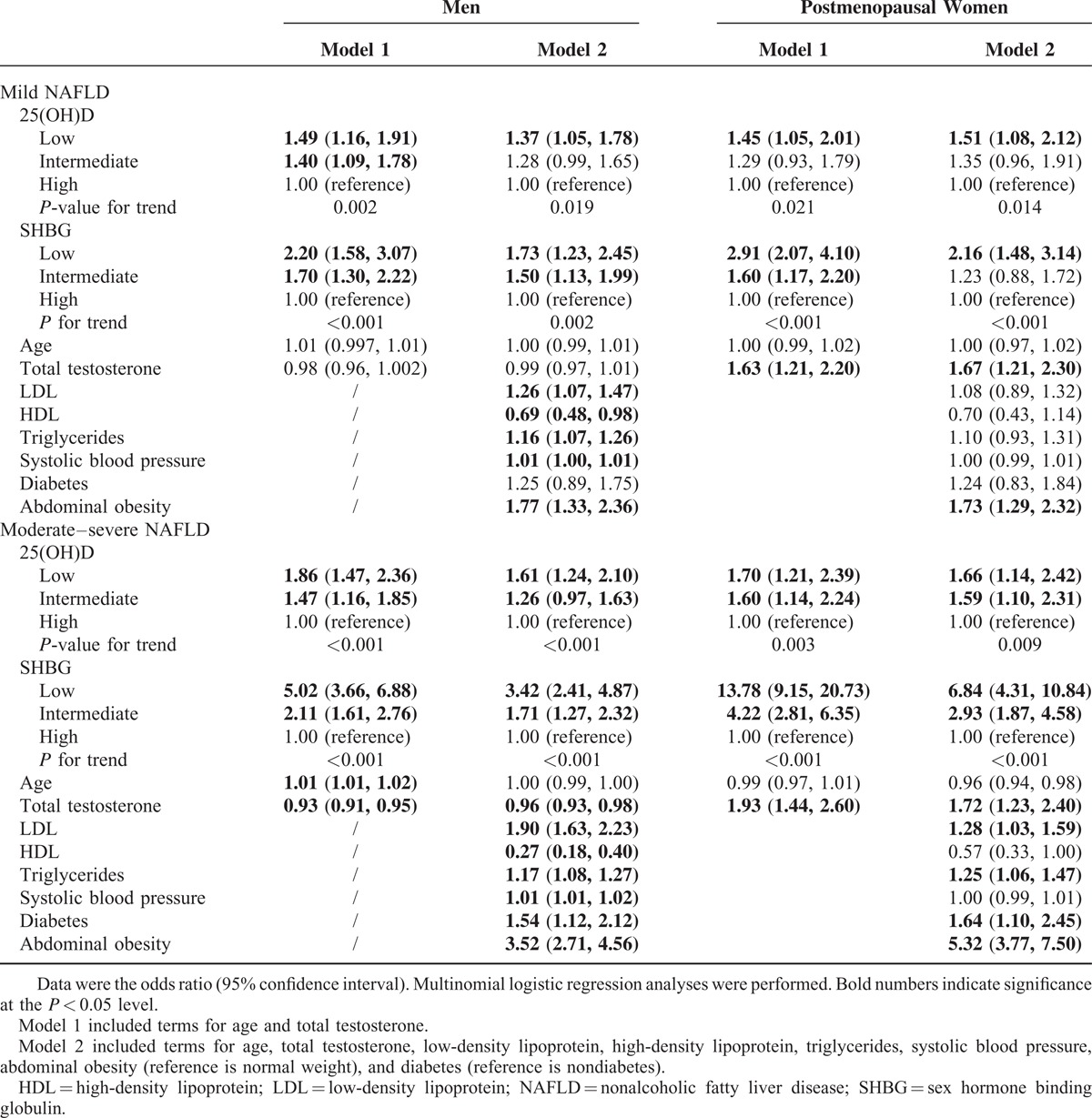
Separate Associations of 25(OH)D and Sex Hormone Binding Globulin Level With Nonalcoholic Fatty Liver Disease

### The Combined Association of 25(OH)D and SHBG With NAFLD

As shown in Tables 4 and 5, compared with the combination of high 25(OH)D and SHBG, most groups trended toward higher odds of mild and moderate–severe NAFLD, but the combination of low 25(OH)D and SHBG was associated with the highest or almost highest odds of mild NAFLD (OR 3.78, 95% CI 2.30, 6.19, men; OR 3.58, 95% CI 1.99, 6.46, women) and moderate–severe NAFLD (OR 11.08, 95% CI 6.85, 17.92; OR 15.18, 95% CI 8.00, 28.82, women) in model 1. This result was also observed in model 2, although the effect size was smaller.

**TABLE 4:**
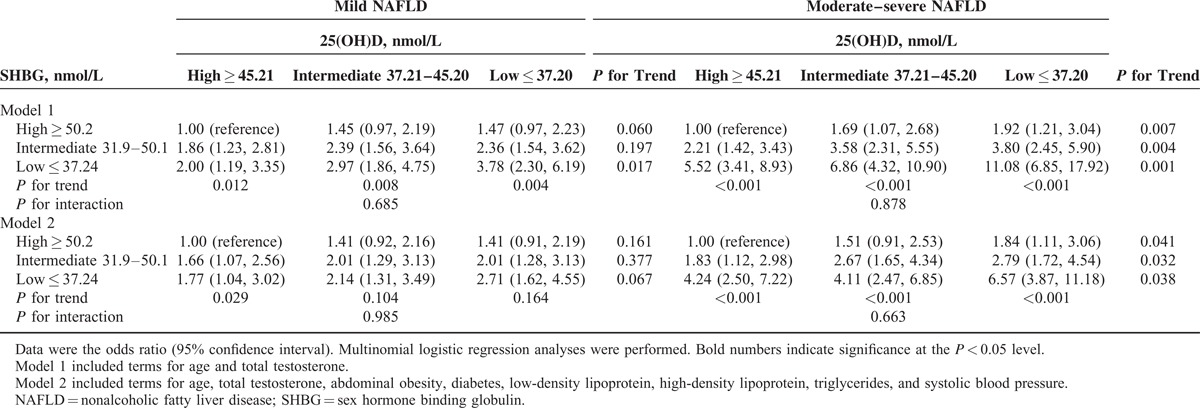
Combined Associations of 25(OH)D and Sex Hormone Binding Globulin Level With Nonalcoholic Fatty Liver Disease in Men

**TABLE 5:**
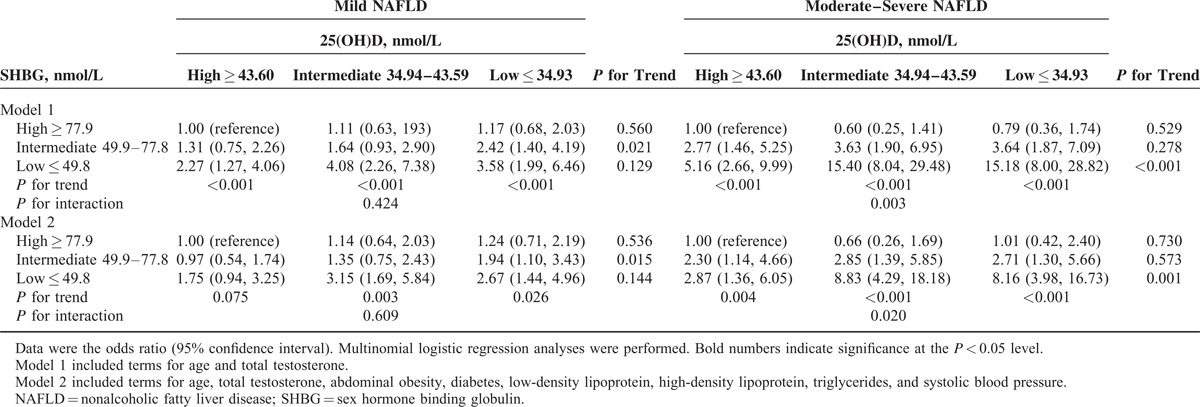
Combined Associations of 25(OH)D and Sex Hormone Binding Globulin Level With Nonalcoholic Fatty Liver Disease in Postmenopausal Women

We found a steep increase in the OR for NAFLD with decreasing SHBG when the 25(OH)D group was held fixed, especially in moderate–severe NAFLD (all *P* for trend <0.01). In contrast, there was relatively less change in the OR for NAFLD with decreasing 25(OH)D when the SHBG group was fixed.

### Sensitivity Analyses

The Spearman correlation between metabolic factors and the 25(OH)D and SHBG levels was analyzed (Supplemental Table S2). The level of 25(OH)D was correlated with ALT, blood pressure, and lipid profile in men and with HDL and triglycerides in postmenopausal women. SHBG was correlated with almost all of the metabolic factors listed in men and women. Additionally, when 25(OH)D and SHBG levels were included as continuous variables, NAFLD ORs were still significant (*P* < 0.05) in moderate–severe NAFLD (Supplemental Table S3). The average age of the men examined was approximately 50 to 60 years, which may be representative of later adulthood, so we also analyzed the association in men younger than 50 (Supplemental Table S4). The association was similar in all men. We further explored the association of SHBG and 25(OH)D in combination with metabolic syndrome (Supplemental Table S5). The combination of low 25(OH)D and low SHBG was still associated with the highest odds of metabolic syndrome.

## DISCUSSION

For the first time, this study examined the combined association of 25(OH)D and SHBG with the risk of mild and moderate–severe NAFLD in men and postmenopausal women. Low levels of SHBG were associated with an elevated risk of NAFLD; however, the combined association of low SHBG and low 25(OH)D was much larger, especially in moderate–severe NAFLD, with a 6.57 times higher risk in men and 8.16 times higher risk in postmenopausal women, suggesting a substantially increased risk of NAFLD for people with low SHBG who also have vitamin D deficiency. The associations were independent of age, total testosterone, abdominal obesity, diabetes, and lipid profile. These results were based on a large sample size and objective measures of anthropometrics and metabolic risk factors.

The findings of the present study, based on comparisons of separate and combined associations of 25(OH)D and SHBG, provided new insight by indicating that the combination of low 25(OH)D and SHBG may be a stronger risk factor against having NAFLD than either factor on its own. The mechanisms underlying this interaction are unclear. In principle, higher levels of vitamin D may strengthen the protective association of higher SHBG, either through independent pathophysiological mechanisms^25–27^ or as a factor that could increase androgen synthesis in men.^28^ Vitamin D and SHBG combinations may also simply represent incremental increases in degrees of chronic inflammation, with the lowest vitamin D/lowest SHBG representing the highest degree of chronic inflammation, and the highest vitamin D/highest SHBG representing the lowest degree of chronic inflammation.

Consistent with our findings, previous studies also found that SHBG was negatively associated with NAFLD.^5,7^ A study has reported that SHBG, but not testosterone, is negatively related to the severe NAFLD.^29^ Another Chinese study also had similar results^30^ after the adjustment for testosterone. This indicates that an intrinsic relationship may exist between SHBG and NAFLD. As a production of the liver,^7^ it is reasonable to deduce that the SHBG production and levels may be affected by the state of health of liver. Selva et al^31^ showed that monosaccharide-induced de novo lipogenesis inhibited human SHBG expression. In human subjects, it is the liver fat, but not visceral fat or total body fat, that was found to be an independent predictor of serum SHBG levels.^7,32^ More importantly, with lifestyle modifications, a decrease in liver fat was strongly correlated with an increase in circulating SHBG in healthy subjects, independent of visceral and total body fat. Considering the strong association of SHBG with NAFLD, whether SHBG is just a marker of NAFLD or has an active role in the development and progression of NAFLD and liver fat accumulation may be a question of great importance in the future.

Given that vitamin D deficiency and NAFLD often coexist, emerging evidence indicates a probable causative association between vitamin D deficiency and NAFLD. A large cross-sectional study including 6567 Koreans found that subjects in the high 25(OH)D tertile levels had a decreased risk for NAFLD independent of BMI and metabolic syndrome.^33^ Other studies reported similar results in European and Australian subjects.^25,26^ Several mechanisms may be involved. Vitamin D acts on the adipocytes, inhibits inflammatory cytokines and increases adiponectin secretion.^34,35^ It may also downregulate the expression of toll-like receptors on liver cells and thus ameliorates inflammation.^27^

Vitamin D and SHBG are also associated in previous studies.^12,13^ Two studies found that 25(OH)D and SHBG were significantly associated in men.^12,36^ Though no study has directly determined if vitamin D supplementation could increase SHBG levels, vitamin D supplementation may be helpful to men who have low testosterone levels. In a randomized controlled trial, Pilz et al found that 3332 IU vitamin D daily supplementation for 1 year increased total, bioavailable, and free testosterone levels,^28^ though another analysis of small clinical trials with short durations revealed that vitamin D supplementation was not related with increased testosterone levels.^37^ Few studies investigated this association in women in general, but one study in women with polycystic ovary syndrome also reported that there was a significant association between vitamin D deficiency and SHBG.^38^ We also observed a steep increase in OR for moderate–severe NAFLD with decreasing SHBG when each 25(OH)D group was held fixed, but not with decreasing 25(OH)D when the SHBG group was fixed. This indicate that SHBG may have a greater impact on this association than 25(OH)D, which is in agreement with a previous comment that “the crosstalk between inflammation, T2D, sex steroids, and the risk for CVD seems to converge on a reduction in the levels of SHBG.”^7^ NAFLD may also be added to this crosstalk.

This study had some strengths. First, it is the first study with a relatively large sample size to explore the combined association of 25(OH)D and SHBG with NAFLD. Second, it had strong quality control because the same trained staff completed data collection at every study site. Third, our data source is from a general population as opposed to a clinic-based population, so the findings may be more accurately representative. However, there were some limitations of this study as well. First, because of the cross-sectional design, we could not obtain a causal relationship among 25(OH)D, SHBG, and NAFLD. Second, the use of liver ultrasonography has certain limitations. However, liver biopsy is not feasible in such a large sample. Meanwhile, numerous epidemiological studies use ultrasonography to diagnose fatty liver.^21,30,39^ Saadeh et al's^22^ criteria to diagnose fatty liver could provide up to 93% sensitivity with a positive predictive value of 62% for the histological diagnosis of NAFLD. Therefore, ultrasonography may be a relatively feasible method with acceptable sensitivity and specificity in large epidemiological studies. However, more studies may use liver biopsy to confirm the causal relationship in the future. Finally, we did not test for viral hepatitis antibody, especially the hepatitis C virus. The exclusion of viral hepatitis on the basis of self-report may have a recall and information bias. However, the prevalence of chronic hepatitis C is low in China. As shown by a recent national survey, the prevalence rate of anti-HCV is only 0.43% in mainland China.^40^

In conclusion, the negative associations of high 25(OH)D and high SHBG levels with NAFLD are strongest when viewed in combination in men and postmenopausal women. Further studies should determine the cause–effect relationship and investigate the underlying mechanisms. Whether NAFLD is best prevented by improving levels of both 25(OH)D and SHBG levels may require further examination.

## Supplementary Material

Supplemental Digital Content
