# Supplementary material for: Combined Association of Vitamin D and Sex Hormone Binding Globulin With Nonalcoholic Fatty Liver Disease in Men and Postmenopausal Women: A Cross-Sectional Study
Source: Medicine (Baltimore). 2016 Jan 29;95(4):e2621. doi: 10.1097/MD.0000000000002621 (PMC5291588; doi:10.1097/MD.0000000000002621)
Supplement: Supplemental Digital Content [file medi-95-e2621-s001.doc]

Supplemental Table S1 Characteristics of participants by sex (N=4150)

| Characteristic | Men | Postmenopausal Women | Age-adjusted *P* |
| --- | --- | --- | --- |
| *N* | 2689 | 1461 |  |
| Age, years | 52(13) | 64(7) |  |
| Diabetes, % | 12.9 | 16.6 | .613 |
| NAFLD, % |  |  |  |
| Mild | 22.0 | 22.6 | .870 |
| Moderate-severe | 32.1 | 25.9 | .180 |
| SHBG, nmol/L | 45.6(24.9) | 69.2(33.8) | <.001 |
| 25(OH)D, nmol/L | 43.06(11.52) | 40.53(10.48) | <.001 |
| ALT, IU/L | 26(19) | 21(12) | <.001 |
| SBP, mmHg | 132(20) | 138(21) | .981 |
| DBP, mmHg | 80(12) | 79(12) | <.001 |
| LDL, mmol/L | 2.93(0.69) | 3.12(0.75) | <.001 |
| HDL, mmol/L | 1.37(0.32) | 1.53(0.33) | <.001 |
| Triglycerides, mmol/L | 1.91(1.97) | 1.71(1.23) | .684 |
| Total testosterone, nmol/L | 16.1(5.6) | 0.6(0.5) | <.001 |
| Body mass index, kg/m^2^ | 24.5(3.3) | 24.7(3.6) | .105 |
| Waist circumference, cm | 83(9) | 80(10) | <.001 |

Values are the mean (SD) unless otherwise noted.

ALT, alanine aminotransferase; DBP, diastolic blood pressure; HDL, high density lipoprotein; LDL, low density lipoprotein; SHBG, sex hormone binding globulin; SBP, systolic blood pressure.

Supplemental Table S2 Spearman correlation coefficients between metabolic factors and 25(OH)D or SHBG in men and postmenopausal women

|  | Men | |  | Postmenopausal women | |
| --- | --- | --- | --- | --- | --- |
|  | 25(OH)D | SHBG |  | 25(OH)D | SHBG |
| 25(OH)D | / | **0.186** |  | / | **0.062** |
| Age | **0.251** | **0.510** |  | **-0.056** | **0.243** |
| ALT | **-0.095** | **-0.260** |  | -0.019 | **-0.218** |
| SBP | **0.045** | **0.067** |  | -0.035 | **-0.117** |
| DBP | -0.023 | **-0.097** |  | -0.029 | **-0.170** |
| LDL | **-0.104** | **-0.082** |  | 0.016 | -0.034 |
| HDL | **0.070** | **0.320** |  | **0.062** | **0.290** |
| Triglycerides | **-0.253** | **-0.373** |  | **-0.143** | **-0.331** |
| Total testosterone | **0.110** | **0.599** |  | 0.040 | **-0.078** |
| Body mass index | -0.022 | **-0.356** |  | -0.010 | **-0.431** |
| Waist circumference | 0.001 | **-0.280** |  | 0.025 | **-0.355** |

Data were spearman correlation coefficients. Bold number indicates significance at the 0.05.

ALT, alanine aminotransferase; DBP, diastolic blood pressure; HDL, high density lipoprotein; LDL, low density lipoprotein; SHBG, sex hormone binding globulin; SBP, systolic blood pressure.

Supplemental Table S3 associations of 25(OH)D and SHBG with non-alcoholic fatty liver disease

|  | Men | |  | Postmenopausal women | |
| --- | --- | --- | --- | --- | --- |
|  | Model 1 | Model 2 |  | Model 1 | Model 2 |
| Mild NAFLD |  |  |  |  |  |
| 25(OH)D | **0.99 (0.98, 0.99)** | 0.99(0.98, 1.00) |  | **0.99(0.97, 1.00)** | 0.99(0.97, 1.00) |
| SHBG | **0.98 (0.98, 0.99)** | **0.99(0.98, 0.99)** |  | **0.99(0.98, 0.99)** | **0.99(0.99, 1.00)** |
| Moderate-severe NAFLD |  |  |  |  |  |
| 25(OH)D | **0.98(0.97, 0.98)** | **0.98 (0.97, 0.99)** |  | **0.98(0.97, 0.99)** | **0.98(0.97, 1.00)** |
| SHBG | **0.96(0.95, 0.97)** | **0.97 (0.96, 0.98)** |  | **0.96(0.96, 0.97)** | **0.97(0.97, 0.98)** |

Data were odds ratio (95% confidence interval). Multinomial logistic regression analyses were performed. Bold number indicates significance at the 0.05. NAFLD, non-alcoholic fatty liver disease; SHBG, sex hormone binding globulin.

Model 1 included terms for age, total testosterone.

Model 2 included terms for age, total testosterone, abdominal obesity, diabetes, low-density lipoprotein, high-density lipoprotein, triglycerides and systolic blood pressure.

Supplemental Table S4 Combined associations of 25(OH)D and sex hormone binding globulin level with non-alcoholic fatty liver disease in men younger than 50 years old

|  | **Men <50yrs** | | |  |
| --- | --- | --- | --- | --- |
|  | 25(OH)D, nmol/L | | |  |
| SHBG, nmol/L | High  (≥42.96) | Intermediate  (35.81-42.95) | Low  (≤35.80) | *P* for trend |
| High (≥37.5) | 1.00 (reference) | **2.43(1.32, 4.46)** | 1.07(0.59, 1.94) | 0.004 |
| Intermediate(24.9-37.4) | **1.88(1.04, 3.41)** | **2.48(1.39, 4.43)** | **5.66(2.94, 10.92)** | 0.001 |
| Low (≤24.8) | **3.89(2.07, 7.32)** | **3.92(2.07, 7.42)** | **6.58(3.34, 12.99)** | 0.063 |
| *P* for trend | <0.001 | 0.402 | <0.001 |  |
| *P* for interaction |  | 0.253 |  |  |

The model included terms for age, total testosterone, abdominal obesity, diabetes, low-density lipoprotein, high-density lipoprotein, triglycerides and systolic blood pressure. Mild and moderate-severe NAFLD was combined into total. Bold number indicates significance at the 0.05.

Supplemental Table S5 Combined associations of 25(OH)D and sex hormone binding globulin level with metabolic syndrome in men and postmenopausal women

|  | 25(OH)D, nmol/L | | | *P* for |
| --- | --- | --- | --- | --- |
| SHBG, nmol/L | High | Intermediate | Low | trend |
| **Men** |  |  |  |  |
| High | 1.00 (reference) | 0.59(0.33, 1.08) | 0.82(0.47, 1.45) | 0.462 |
| Intermediate | 0.85(0.50, 1.44) | **1.67(1.03, 2.71)** | **1.90(1.17, 3.09)** | 0.002 |
| Low | **2.78(1.63, 4.73)** | **3.06(1.85, 5.07)** | **3.56(2.14, 5.95)** | 0.291 |
| *P* for trend | 0.001 | <0.001 | <0.001 |  |
| *P* for interaction |  | 0.241 |  |  |
| **Postmenopausal women** |  |  |  |  |
| High | 1.00 (reference) | 0.99(0.54, 1.81) | 0.80(0.44, 1.47) | 0.507 |
| Intermediate | **2.56(1.51, 4.34)** | **2.71(1.56, 4.68)** | **2.74(1.59, 4.69)** | 0.738 |
| Low | **5.07(2.92, 8.80)** | **7.78(4.57, 13.25)** | **7.89(4.66, 13.35)** | 0.074 |
| *P* for trend | <0.001 | <0.001 | <0.001 |  |
| *P* for interaction |  | 0.048 |  |  |

Data were odds ratio (95% confidence interval). Multinomial logistic regression analyses were performed. Bold number indicates significance at the 0.05.

The model included terms for age and total testosterone.
